# Switching the unidirectional reflectionlessness by polarization in non-ideal PT metamaterial based on the phase coupling

**DOI:** 10.1038/s41598-017-11376-w

**Published:** 2017-09-06

**Authors:** Ruiping Bai, Cong Zhang, Xintong Gu, Xing Ri Jin, Ying Qiao Zhang, YoungPak Lee

**Affiliations:** 1 Department of Physics, College of Science, Yanbian University, Yanji, Jilin, 133002 China; 2 Quantum Photonic Science Research Center and Department of Physics, Hanyang University, Seoul, 133-791 Republic of Korea

## Abstract

An effective scheme on switching the exceptional point(EP) where unidirectional reflectionlessness occurs is firstly proposed in non-ideal PT metamaterial via the polarization of incident light. The unidirectional reflectionlessness could be effectively controlled only by adjusting the phase coupling of the two resonators which are consisted of two identical but vertically placed crosses and are excited by incident light as an effective gain. Besides, the unidirectional perfect absorber occurs in the vicinity of EP.

## Introduction

Parity-Time(PT) symmetry theory has attacted much attention since it was proposed by Bender and his colleagues in 1998^1^. At first PT theory was proposed in quantum mechanics, it was considered to be an interesting mathematical discovery without practical application. However, with the development of PT-symmetric media, it has been a hot field in the research of experimental and theoretical physics, which could be applied in optical waveguides^2–5^, perfect cavity absorber lasers^6, 7^, microwave cavities and resonators^8–10^, especially in metamaterials^11–18^ with extraordinary properties^19^. An ideal PT-symmetric system is an open physical system with strictly balanced gain and loss distributions, which can be typically represented by a pair of coupled optical waveguides^20^. It opens a new avenue for exploring the extraordinary physics of non-Hermitian Hamiltonians. The non-Hermitian Hamiltonians with PT-symmetry could show entire real eigenvalue spectrum below a phase transition point. That is to say, such systems can suffer a phase transition from the PT-symmetric phase to the PT broken phase when certain external parameters exceed a critical value. After the phase transition point, the corresponding energy spectra change from real to complex dramatically. A PT media in optical structure can give rise to a wide range of counterintuitive phenomena such as a single-mode laser^10^, power oscillation violating left-right symmetry^20^, loss-induced transparency^21^, novel beam refraction^22^, optical solitons^23, 24^, spectral signature^25^, and unidirectional reflectionlessness^11, 26–28^. In optical waveguide system, Feng *et al*.^11^ experimentally demonstrated an unidirectional reflectionless PT metamaterial near the phase transition point at optical frequencies in 2013, in which the reflection from one side was significantly diminished. Recently, Fu *et al*.^29^ demonstrated zero-index metamaterials with parity-time (PT) symmetry can be achieved by introducing defects with loss/gain inside epsilon-near zero media. The phenomenon of unidirectional transparency at EP took place well in their PT symmetric systems. Sun *et al*.^17^ demonstrated that the coherent perfect absorber(CPA) using the passive resonators was excited by incident wave as an effective gain in 2014. Besides, in layered periodic structure with balanced gain and loss, the property of PT-symmetry has been the subject of extensive studies^28, 30, 31^, while there are few reports about it in periodically-distributed loss metamaterials.

In this paper, we present a non-ideal PT metamaterial structure to achieve polarization switching of unidirectional reflectionless phenomena based on Fabry-Pérot(FP) resonance only by tuning the structural parameter *d*_2_, inspired by ref. 17 that only the passive resonators are excited by incident waves as an effective gain. The unidirectional reflectionless phenomenon at EP can be realized by altering the polarization angle of incident wave in either side of the structure. Meanwhile, the polarization angle in a wide range of nearly ±15° is valid to show the excellent unidirectional reflectionless phenomena as well.

## Results

**Figure 1:**
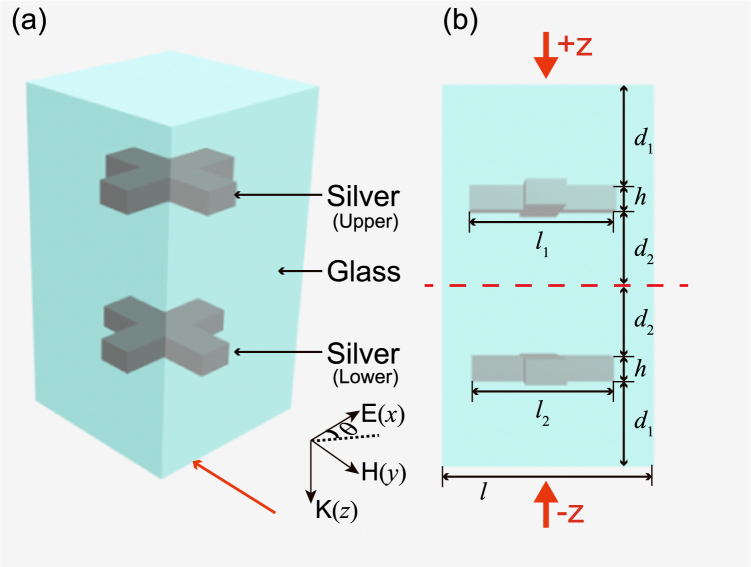
(**a**) Schematic of the proposed the metamaterial structure. (**b**) The front view (indicated by red arrow above) of the structure. The geometric parameters are *h* = 20 nm, *l*_1_ = 220 nm, and *l*_2_ = 210 nm. The *d*_1_ varies with *d*_2_. The vertical height of unit cell satisfies 2*d*_1_ + 2*d*_2_ + 2*h* = 800 nm. The periods in both *x* and *y* directions are *l* = 400 nm. The incident radiation is along + *z* direction or −*z* direction and the polarization of them is both along *x* direction. Here, the polarization angle is marked by *θ*, that is, *θ* = 0(*θ* = 90°) represent the electric field along *x*(*y*) direction.

The unit cell of the non-ideal PT metamaterial structure is depicted in Fig. 1(a) and (b). The structure consists of upper and lower identical silver crosses with the same width (60 nm) but different lengths in *x* and *y* directions, and both of them are embedded in glass cladding with permittivity 2.25. The lower silver cross (LSC) is rotated 90 degrees clockwise or anticlockwise with respect to upper silver cross (USC). The polarization angle is marked by *θ* in Fig. 1, that is to say, polarization angle *θ* = 0(*θ* = 90°) represents the electric field along *x*(*y*) direction. The permittivity of silver is complied with Drude model with the plasmon frequency *ω*_*pl*_ = 1.366 × 10^16^ rad/s and the damping constant *ω*_*c*_ = 3.07 × 10^13^s^−1^ ^32, 33^. The numerical calculation is carried out by using the commercial finite difference time domain software package (CST Microwave Studio). The boundary conditions in the CST settings are the unit cell in *x* and *y* directions, while is open in *z* direction. The mesh type is tetrahedral.

**Figure 2:**
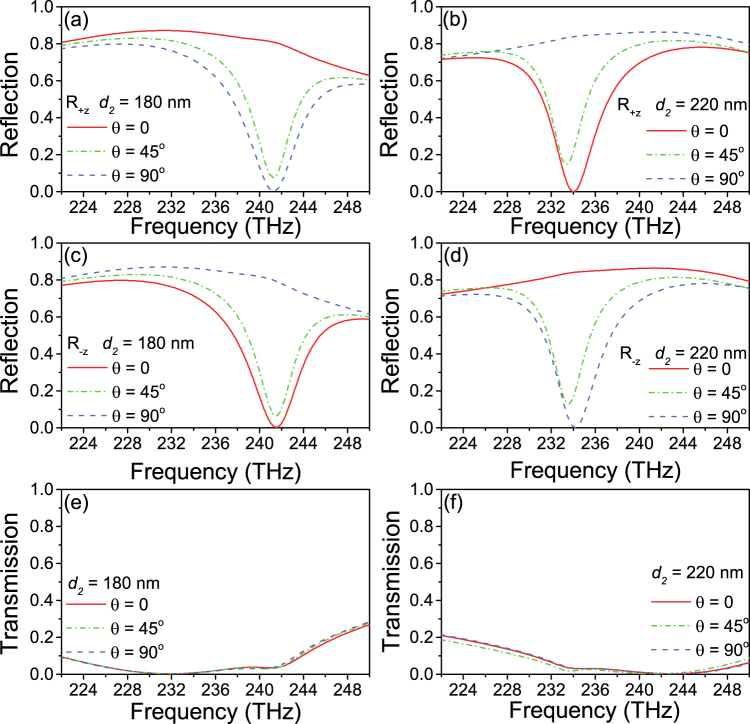
Reflection (**a**–**d**) and transmission (**e**-**f**) spectra for the +*z* direction and −*z* direction when *d*_2_ = 180 nm and *d*_2_ = 220 nm under the change of *θ*.

Figure 2 shows the reflection (Fig. 2(a–d)) and transmission (Fig. 2﻿(e,f)) spectra versus incident wave in +*z* (Fig. 2(a,b)) and −z (Fig. 2(c,d)) directions under *θ* = 0,45°,90°, respectively, when the distance *d*_2_ is 180 nm and 220 nm. For the case of *d*_2_ = 180 nm, Fig. 2(a) and (c) can unambiguously see that the high reflection spectra of *θ* = 0 (low reflection spectra of *θ* = 90°) in + *z* incident radiation is exactly the same with the *θ* = 90° (*θ* = 0) in −*z* incident radiation. With regard to the case of *d*_2_ = 220 nm, the result is similar. It is obvious that our design can be used for a polarization switching which can determine the high reflectance or low reflectance. Furthermore, from Fig. 2(e) and (f), the transmission spectra are identical in −*z* and + *z* directions. For *d*_2_ = 180 nm and *d*_2_ = 220 nm, the transmittances are both very low and nearly invariable in different *θ*.

**Figure 3:**
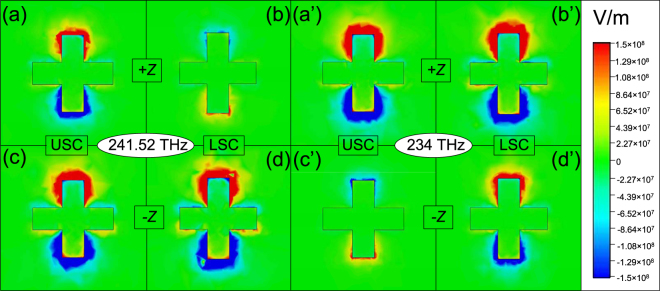
The *z*-component distributions of electric field of the two crosses for both +*z* and −*z* directions at 241.52 THz (**a**-**d**) and 234 THz (**a**′-**d**′), respectively, when *d*_2_ is 180 nm and 220 nm.

Subsequently, in order to reveal the potential physics in our structure, we calculate the electric field distributions of USC and LSC in *z*-component for a normally incident plane wave with *θ* = 0 at two EPs. The electric field distributions of the first EP with a frequency 241.52 THz in the case of *d*_2_ = 180 nm are shown in Fig. 3(a–d), and that of the second EP with a frequency 234 THz in *d*_2_ = 220 nm are shown in Fig. 3(a'-d'). For the first EP, the USC is strongly excited (Fig. 3(a)) but the LSC is weakly excited (Fig. 3(b)) by the +*z* incident radiation, and their directions of induced currents are opposite. This means that the phase difference of them nearly *π* will lead to a high reflection peak (the peak value close to 0.8, see Fig. 2(a) red solid line). Instead, the USC and LSC are both strongly excited by the −*z* incident wave (Fig. 3(c) and (d)). Their induced currents are in same directions. The phase difference of them is nearly 2*π* so that the peak value verges on 0 (see Fig. 2(c) red solid line). The next, looking at the second EP, the results are quite opposite. The phase difference of induced currents between USC and LSC are $\sim 2\pi$ in +*z* direction (Fig. 3(a’) and (b’)) and $\sim \pi$ in −*z* direction (Fig. 3(c’) and (d’)), which correspond to the low reflection peak (the peak value is ∼0, Fig. 2(b) red solid line) and high reflection peak (the peak value is ∼0.84, Fig. 2(d) red solid line). In both cases, the contrast ratio of reflection extremely approaches to 1 in different incident directions. That is to say, our design can tremendously achieve bilateral unidirectional reflectionless phenomena at two EPs, which based on FP resonance by the USC and LSC coupling. Besides, the high and low reflection spectra counterchange in two EPs.

**Figure 4:**
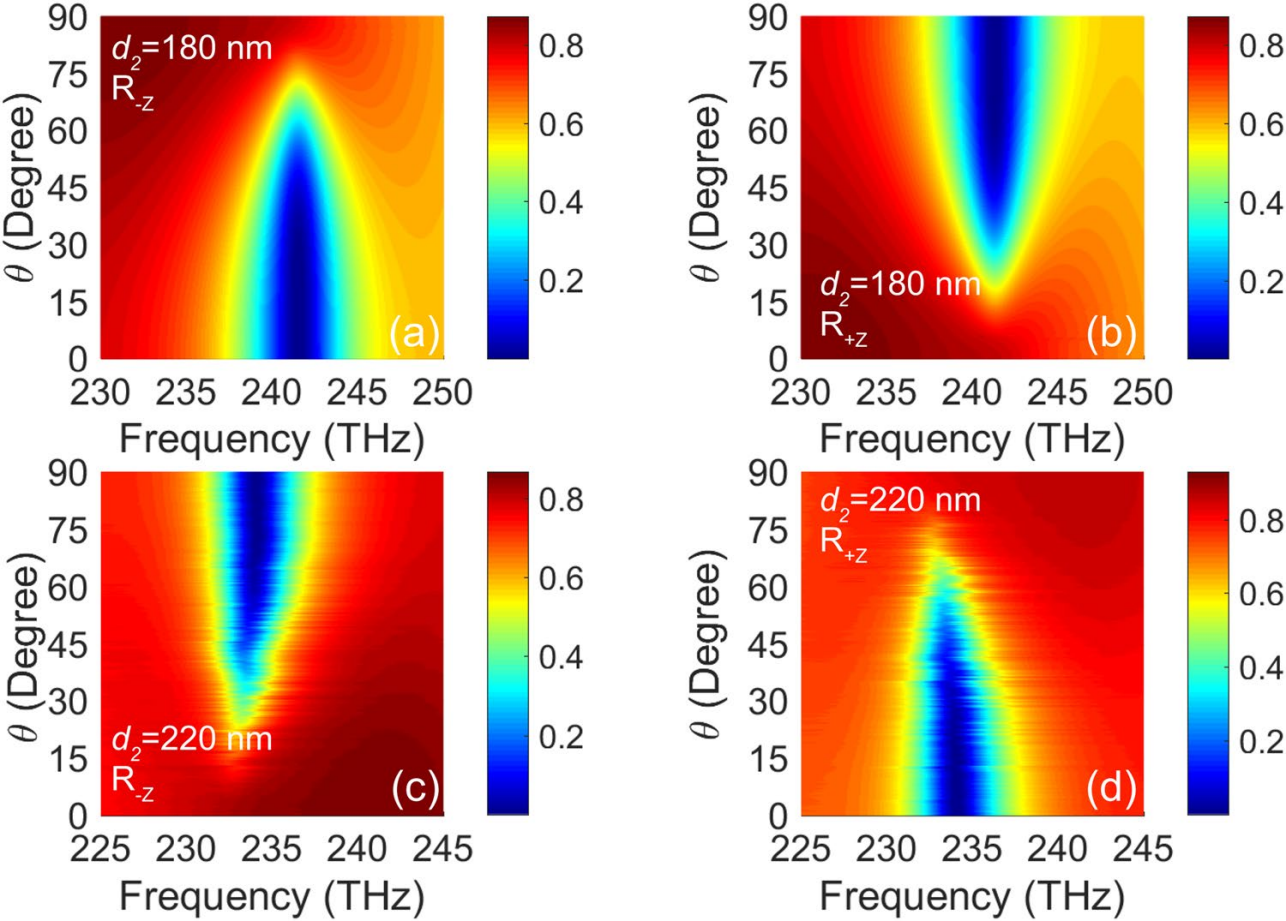
Dependence of the reflection on the frequency of incident wave and polarization angle of 0 in +*z* and −*z* incident wave at *d*_2_ = 180 nm ((**a**) and (**b**)) and *d*_2_ = 220 nm ((**c**) and (**d**)), respectively.

Then we concentrate on the feature of polarization switching to interpret the statements. Figure 4(a–d) show the reflectances at two EPs as a function of the *θ* and the frequency of incident wave in +*z* ((b) and (d)) and −*z* ((a) and (c)) directions. In the case of EP in *d*_2_ = 180 nm, from Fig. 4(a) and (b), we can clearly see that the unidirectional reflectionless phenomenon appears at 241.52 THz with the increase of *θ* from 0 to 90°. More remarkably, the *θ* in a wide range of nearly ±15° can realize the unidirectional reflectionless phenomena. With increase of *θ*, the reflectance increases (decreases) in −*z* (+*z*) direction at 241.52 THz, which exactly indicate that the structure can be used for a polarization switching to determine the high reflectance or low reflectance. For the other EP in *d*_2_ = 220 nm, the unidirectional reflectionless phenomenon appears in the vicinity of 234 THz with *θ* increased from 0 to 90° (see Fig. 4(c,d)). The resonant peak position is related to the detuning of USC in *x* direction and LSC in *y* direction. For the case of *d*_2_ = 180 nm, the detuning 0.08 THz is lower than 0.48 THz in *d*_2_ = 220 nm, which cause the good stability of the resonant frequency. Actually, the detuning of USC in *x* direction and LSC in *y* direction are required to be consistent in our design for the sake of polarization switching. Likewise, at the EP in *d*_2_ = 220 nm, we can not only realize polarization switching but also provide a pretty clearly unidirectional reflectionless phenomenon in the range of nearly ±15°.

Now, we adopt the transfer matrix methods to further analyze the scattering properties and verify the related phenomena at EPs in our system. In fact, there is a close similarity between electromagnetic scattering and quantum mechanical^34^. Based on the similarity between the Hamiltonian **H** in an open quantum system and the scattering matrix **S** in Maxwell’s framework^35, 36^, the optical properties of our non-ideal PT metamaterial system can be simply described by **S** as follows

1 $$(\begin{array}{c}{A}_{+}\\ {A}_{-}\end{array})={\bf{S}}(\begin{array}{c}{B}_{+}\\ {B}_{-}\end{array})(\begin{array}{cc}t & {r}_{-{\rm{z}}}\\ {r}_{+{\rm{z}}} & t\end{array})(\begin{array}{c}{B}_{+}\\ {B}_{-}\end{array}),$$

where *A*_±_ (*B*_±_) are the complex electric field amplitudes of the outgoing waves (incoming waves) in the +*z* and −*z* directions, respectively. *r*_+z,−z_ and *t* are the complex reflection and transmission coefficients for the incident radiation in the +*z* and −*z* directions, respectively. *r*_+z,−z_ and *t* are related to the entries of transfer matrix **M**, which according to

2 $${r}_{+{\rm{z}}}=\frac{-{M}_{21}}{{M}_{22}},\,{r}_{-{\rm{z}}}=\frac{{M}_{12}}{{M}_{22}},\,t=\frac{1}{{M}_{22}}.$$

The expression for our whole non-ideal PT metamaterial system of transfer matrix (**M**_*all*_) can be obtained by multiplying the transfer matrices of the individual components^33, 37^

3 $${{\bf{M}}}_{all}={{\bf{M}}}_{s}^{1}\times {{\bf{M}}}_{p}\times {{\bf{M}}}_{s}^{2}=(\begin{array}{cc}{M}_{11} & {M}_{12}\\ {M}_{21} & {M}_{22}\end{array}),$$

where

4 $${{\bf{M}}}_{s}^{\mathrm{1(2)}}=(\begin{array}{cc}1-\frac{i{\gamma }_{\mathrm{1(2)}}}{\omega -{\omega }_{\mathrm{1(2)}}+i{{\rm{\Gamma }}}_{\mathrm{1(2)}}\mathrm{/2}} & \frac{i{\gamma }_{\mathrm{1(2)}}}{\omega -{\omega }_{\mathrm{1(2)}}+i{{\rm{\Gamma }}}_{\mathrm{1(2)}}/2}\\ -\frac{i{\gamma }_{\mathrm{1(2)}}}{\omega -{\omega }_{\mathrm{1(2)}}+i{{\rm{\Gamma }}}_{\mathrm{1(2)}}\mathrm{/2}} & 1+\frac{i{\gamma }_{\mathrm{1(2)}}}{\omega -{\omega }_{\mathrm{1(2)}}+i{{\rm{\Gamma }}}_{\mathrm{1(2)}}/2}\end{array}),$$

5 $${{\bf{M}}}_{p}=(\begin{array}{cc}{e}^{i\varphi } & 0\\ 0 & {e}^{-i\varphi }\end{array}).$$

Here, ${{\bf{M}}}_{s}^{\mathrm{1(2)}}$ and **M**_*p*_ are the transfer matrices for USC (LSC) and for the phase shift of wave propagating from USC to LSC. *ω*_1(2)_ and Γ_1(2)_ are the resonant frequencies and dissipative losses for the USC (LSC), respectively. *γ*_1(2)_ is the width of the resonance in the USC (LSC) coupled with the incident wave. *ϕ* Is the accumulated phase shift for wave propagating from USC to LSC. *ω* is the frequency of incident wave.

**Figure 5:**
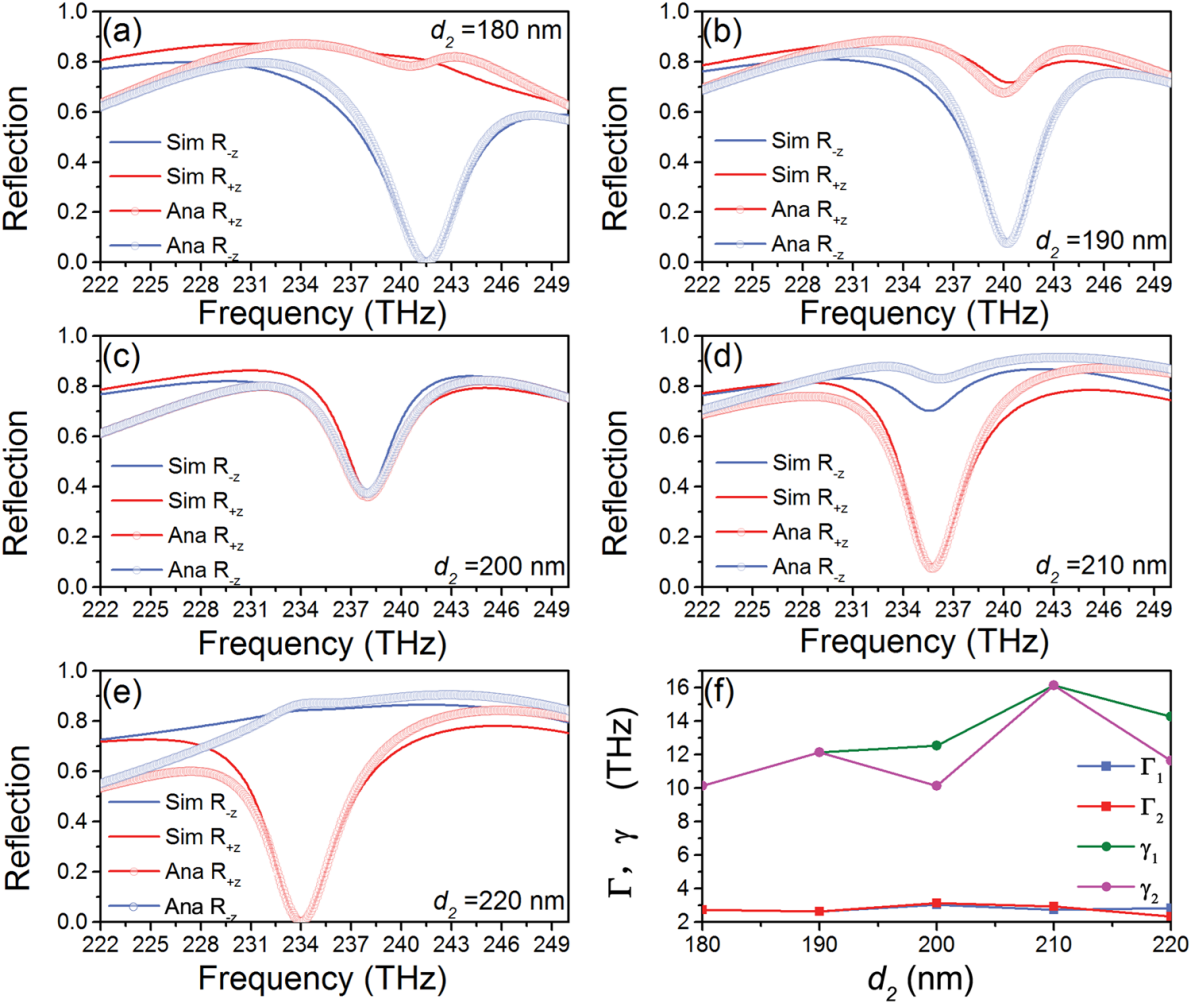
(**a**–**e**) Dependances of simulated and analytical reflection spectra on the parameter *d*_2_ for incident wave with polarization angle of 0. (**f**) The relevant parameters *γ*_1(2)_ and Γ_1(2)_ are obtained from scattering matrix, respectively.

Figure 5(a–e) depict the simulated (red and blue solid lines) and analytical (red and blue lines with circle symbol) reflection spectra in +*z* and −*z* incident directions with the increase of *d*_2_ from 180 nm to 220 nm every 10 nm, respectively. From Fig. 5, we can see that the simulated results are in good agreement with analytical results. The reflectances of +*z* and −*z* directions in *d*_2_ = 180 nm(220 nm) are ∼0.8 and ∼0(∼0 and ∼0.84), respectively, as shown in Fig. 5(a) and (e). The contrast ratios of both them are very close to 1, which excellently indicate that bilateral unidirectional reflectionless phenomena are realized. The same reflectances (near 0.36) in both directions are described in Fig. 5(c). As can be seen from Fig. 5(a) to (e), with the increase of *d*_2_, the high reflection spectra in +*z* direction gradually decline and low reflection spectra in −*z* direction gradually raise. Obviously, the reflections go into reverse in bilateral directions. Parameters *γ*_1(2)_ and Γ_1(2)_ getting from the analytical method are shown in Fig. 5(f). Γ_1(2)_ basically remain invariant while *γ*_1(2)_ has a slight deviation between *d*_2_ = 200 nm and 220 nm.

What’s more, the phase shift *ϕ*_1(2)_ for USC (LSC) can be obtained by Eq. (1) in +*z* and −*z* incident directions, respectively. The phase shift *ϕ*_1(2)_ in +*z* direction is shown as following^33^

6 $$\begin{array}{rcl}{\varphi }_{\mathrm{1(2)}} & = & \arctan [{\rm{Im}}({M}_{s\mathrm{,21}}^{\mathrm{1(2)}}/{M}_{s\mathrm{,22}}^{\mathrm{1(2)}})/{\rm{Re}}({M}_{s\mathrm{,21}}^{\mathrm{1(2)}}/{M}_{s\mathrm{,22}}^{\mathrm{1(2)}})]\\  & = & (\omega -{\omega }_{\mathrm{1(2)}})/({\gamma }_{\mathrm{1(2)}}+{{\rm{\Gamma }}}_{\mathrm{1(2)}}).\end{array}$$

The phase difference *ϕ*_*all*_ between USC and LSC consists of three parts: the respective phase shifts of USC and LSC together with the phase shift for wave propagation from USC to LSC. Therefore, *ϕ*_*all*_ in +*z* direction is equal to *ϕ*_1_ − *ϕ*_2_ + 2*ϕ*, whereas *ϕ*_*all*_ is equal to *ϕ*_2_ − *ϕ*_1_ + 2*ϕ* in −*z* direction. According to Eq. (1) and relevant parameters from the transfer matrix method (Fig. 5(f)), we can easily obtain that the both *ϕ*_*all*_ at two EPs are almost 2*π* by calculation in the positions with reflectance ∼0. This corresponds to the statements in previous section that the induced currents of USC and LSC are in the same directions, which mean the phase difference approaching 2*π* between USC and LSC in both +*z* and −*z* incident directions (Fig. 3).

**Figure 6:**
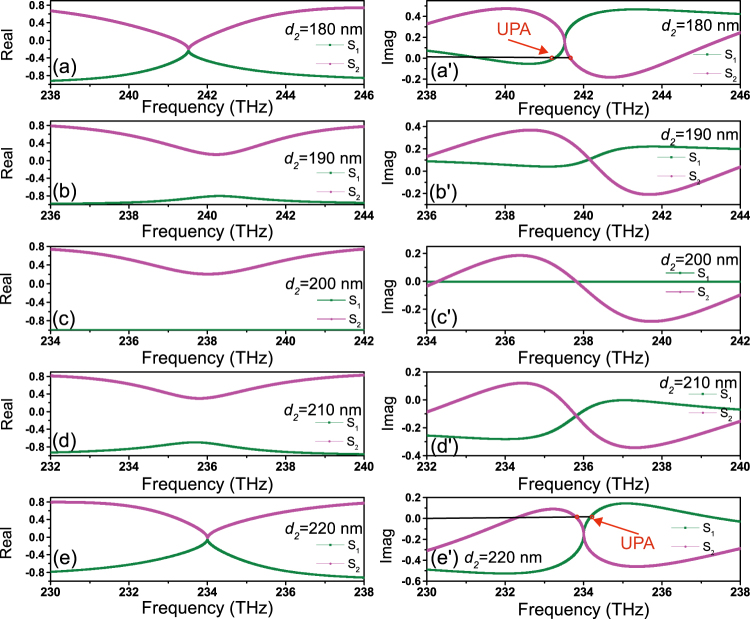
The real and imaginary parts of the eigenvalues for the scattering matrix **S** versus frequency in different *d*_2_.

Hence, we are aimed to illuminate the relevant physics phenomena at two EPs by solving the eigenvalues of **S** in detail. Here, our system is a non-ideal PT system. The complex eigenvalues of **S** are ${S}_{\mathrm{1,2}}=t\pm \sqrt{{r}_{+{\rm{z}}}{r}_{-{\rm{z}}}}$. On the basis of equations (1–5), we plot the real and imaginary part curves of the eigenvalues versus frequency in different *d*_2_ (corresponds to the Fig. 5(a–e)) as depicted in Fig. 6. Looking at the Fig. 6(a)–(a’) and (e)–(e’), it is evident that both the real and imaginary parts of two eigenvalues *S*_1_ and *S*_2_ coincide at one point. We all know that the EP (also related to a non-Hermitian degeneracy of **S**) is a square-root-branch point at mathematics which mean the numerical value under square root is zero at one point, i.e., *r*_+*z*_ *r*_−z_ = 0. On this occasion, the eigenvalues of **S** only have one real part and one imaginary part (nonzero value) respectively correspond to Fig. 6(a)–(a’) and (e)–(e’). That is, EP occurs in our non-ideal PT system. In Fig. 6(c)–(c’), there are two real parts and one imaginary part (zero) at 237.8 THz. At this point, *t* and $\sqrt{{r}_{+z}{r}_{-z}}$ in *S*_1,2_ are both real, so our system is Hermitian. Apart from this point, one of the eigenvalues *S*_1,2_ is real and the other is complex, so *t* and $\sqrt{{r}_{+z}{r}_{-z}}$ in *S*_1,2_ must be both complex. In this case, the imaginary part of *t* are cancelled out by one of the imaginary part of $\sqrt{{r}_{+z}{r}_{-z}}$ in *S*_1,2_. Therefore, our system is non-Hermitian. Then see Fig. 6(b)–(b’) and (d)–(d’), the *S*_1_ and *S*_2_ have one imaginary part (nonzero value) and two real parts when frequency is respectively equal to 240.2 and 235.8 THz. In this case, *t* is complex and $\sqrt{{r}_{+z}{r}_{-z}} > 0$.

Besides that, our metamaterial design can also show unidirectional perfect absorber (UPA) as shown in Fig. 6(a’) and (e’) (see red arrow). The UPA in our design emerges on the vicinity of EP, more extraordinary still, there are two positions where UPA phenomenon occurs. Then, we utilize the distribution of electric field at EP to approximately illustrate the existence of UPA. We can see that the *ϕ*_*all*_ is ~2π in −*z* direction (Fig. 3(c–d)) or +z direction (Fig. 3(a'-b')) from the electric field distribution at EP. Hence, *ϕ*_*all*_ at the two points are nearly equal to 2*π*, which lead to low reflection based on FP resonance. At the same time, because transmission is very low at the two points (see Fig. 2(e) and (f)), absorption approaches to 1 by calculating with formula A = 1 − |*t*|^2^ −|*r*_+*z*,−z_|^2^. Therefore, the UPA is realized in our structure. Additionally, the eigenvalues split in the imaginary part (*r*_+*z*_ *r*_−z_ is complex) at the two points. Therefore, one of the eigenvalues *S*_1,2_ is real and the other is complex. At the two points of UPA near the first EP, the absorptances are both ∼97% with same quality factor of ∼43 when imaginary parts of *S*_1_ or *S*_2_ is 0.(Fig. 6(a’)). While the absorptances of the UPA points near the second EP are ∼92% and ∼93% with quality factor ∼38 and ∼37, respectively, when the imaginary parts of *S*_1_ or *S*_2_ is 0 (Fig. 6(e’)). Consequently, the unidirectional reflectionless phenomenon occurs at the EPs, and UPA is successfully realized as well.

**Figure 7:**
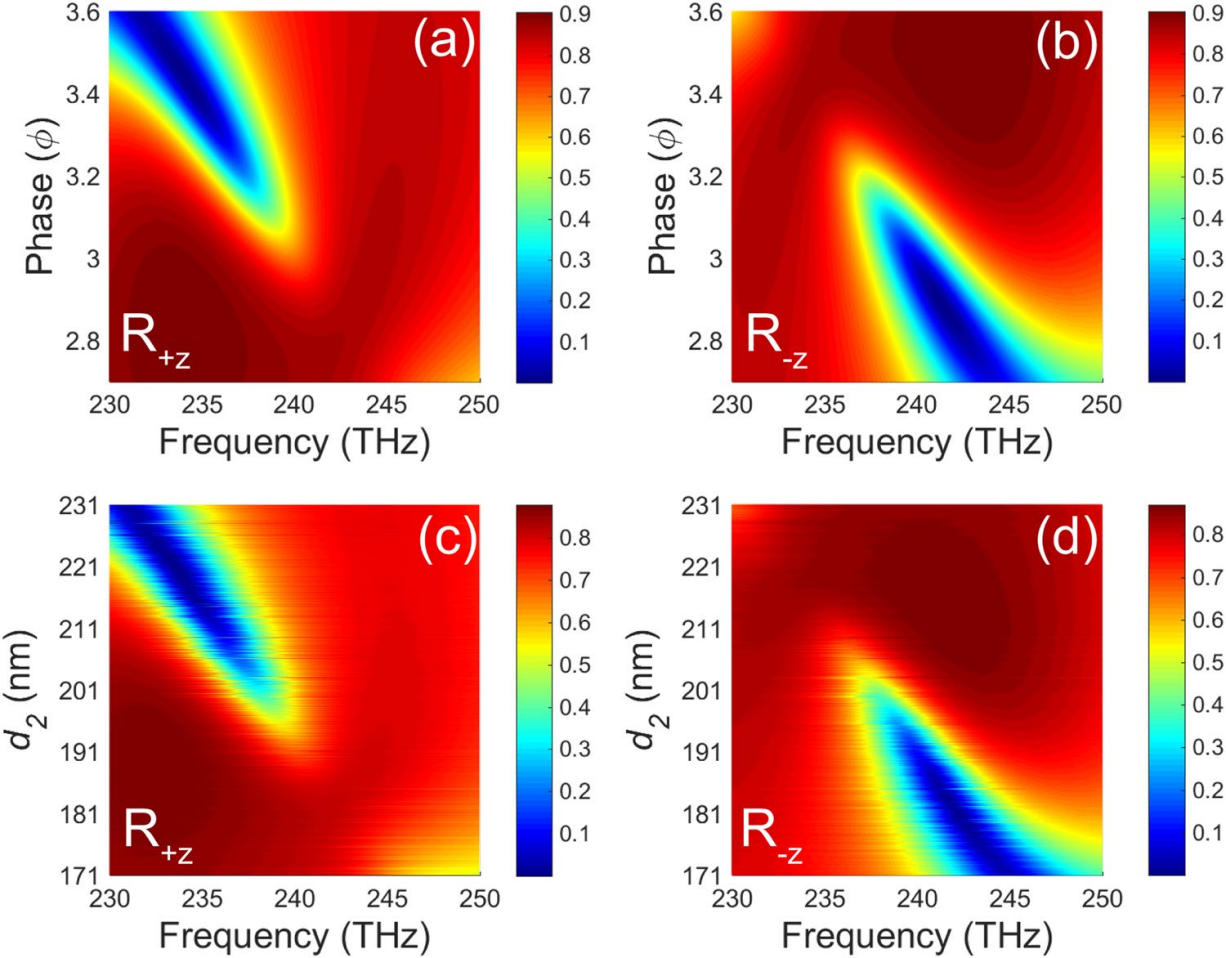
The dispersions of the reflectance peaks of *ϕ* ((**a**) and (**b**)) and *d*_2_ ((**c**) and (**d**)) in +*z* and −*z* incident directions, respectively.

To prove the influence of accumulated phase shift (*ϕ*) and *d*_2_ on bilateral reflections, we also plot the dispersions of the reflectance peaks of them in + *z* and −*z* directions, as shown in Fig. 7(a–d). Comparing Fig. 7(a) with (b) ((c) with (d)), actually we can see that the low reflection region in Fig. 7(a,c) is corresponding to the high reflection region in Fig. 7(b,d) and vice versa. These reveal that the unidirectional reflectionless phenomenon can be effectively achieved in our scheme. Then we pay a close attention to Fig. 7(a,c and b,d), in both incident directions, the dispersions of the reflectance peaks of *ϕ* match very well with *d*_2_. The results show that there are good consistencies between the result of transfer matrix method and result of simulation. Our results are based on the rotational symmetric structure which consists of identical but vertically placed USC and LSC, while in fact the same results can also be achieved as long as the resonant frequency of the upper structure in *x* direction is the same as the lower structure in *y* direction. In the meantime, a small detuning of the resonant frequency in *x* and *y* direction of both upper and lower structures is required.

## Discussion

In conclusion, we have investigated a novel metamaterial structure and showed the switching of EP via the polarization of the incident light in appropriate phase coupling. Interestingly enough, only by appropriately adjusting the phase coupling between two resonators, two UPAs in the vicinity of EP and EPs are clearly observed, respectively. Moreover, the *θ* in the range of nearly ±15° is valid to realize the unidirectional reflectionlessness. These results make a significant platform for realizing the extraordinary properties of non-Hermitian metamaterial system, which possesses potential applications in sensor, filter, isolator, highly compact intergrated and anisotropic nanophotonic devices.
